# Quantitative prediction of integrase inhibitor resistance from genotype through consensus linear regression modeling

**DOI:** 10.1186/1743-422X-10-8

**Published:** 2013-01-03

**Authors:** Koen Van der Borght, Ann Verheyen, Maxim Feyaerts, Liesbeth Van Wesenbeeck, Yvan Verlinden, Elke Van Craenenbroeck, Herman van Vlijmen

**Affiliations:** 1 Tibotec-Virco, Beerse, Belgium

**Keywords:** Consensus model, Genetic algorithm, Integrase, Linear regression, Raltegravir, Resistance

## Abstract

**Background:**

Integrase inhibitors (INI) form a new drug class in the treatment of HIV-1 patients. We developed a linear regression modeling approach to make a quantitative raltegravir (RAL) resistance phenotype prediction, as Fold Change in IC50 against a wild type virus, from mutations in the integrase genotype.

**Methods:**

We developed a clonal genotype-phenotype database with 991 clones from 153 clinical isolates of INI naïve and RAL treated patients, and 28 site-directed mutants.

We did the development of the RAL linear regression model in two stages, employing a genetic algorithm (GA) to select integrase mutations by consensus. First, we ran multiple GAs to generate first order linear regression models (GA models) that were stochastically optimized to reach a goal R^2 ^accuracy, and consisted of a fixed-length subset of integrase mutations to estimate INI resistance. Secondly, we derived a consensus linear regression model in a forward stepwise regression procedure, considering integrase mutations or mutation pairs by descending prevalence in the GA models.

**Results:**

The most frequently occurring mutations in the GA models were 92Q, 97A, 143R and 155H (all 100%), 143G (90%), 148H/R (89%), 148K (88%), 151I (81%), 121Y (75%), 143C (72%), and 74M (69%). The RAL second order model contained 30 single mutations and five mutation pairs (p < 0.01): 143C/R&97A, 155H&97A/151I and 74M&151I. The R^2 ^performance of this model on the clonal training data was 0.97, and 0.78 on an unseen population genotype-phenotype dataset of 171 clinical isolates from RAL treated and INI naïve patients.

**Conclusions:**

We describe a systematic approach to derive a model for predicting INI resistance from a limited amount of clonal samples. Our RAL second order model is made available as an Additional file for calculating a resistance phenotype as the sum of integrase mutations and mutation pairs.

## Background

Recently, new drugs have been developed for the treatment of HIV-1 patients that act at different steps in the viral replication cycle [1,2]. Integrase inhibitors (INIs) target HIV-1 integrase, an enzyme which mediates the integration of HIV-1 viral DNA into the host genome [3,4]. Raltegravir is the first INI approved by the FDA, for use in treatment-naïve and treatment-experienced patients [5,6]. Elvitegravir and S/GSK1349572 are two other INIs in advanced clinical development [7].

Notwithstanding the success of antiretroviral treatment of HIV-1 infection, viral replication cannot always be completely inhibited and this results in the emergence of drug resistance. In clinical practice, resistance testing has proven to be beneficial in designing potent combination regimens. Genotypic tests are preferred to phenotypic tests because of lower cost and faster turnaround time. However, phenotypic tests can provide useful additional information, especially for more complex mutational patterns [8,9]. In this respect, linear regression is successfully applied as a diagnostic service for clinicians, by modeling drug susceptibility as a function of the mutations in the patients viral genome regions that encode for the enzymes HIV-1 protease and reverse transcriptase [10].

In this article, we describe our approach to also generate linear regression models to predict INI resistance from mutations in the integrase (IN) genetic region [11,12]. We show how we applied the methodology for raltegravir (RAL) in deriving a first and second order model on an in-house developed clonal genotype-phenotype database. We report on the performance of both RAL models on four different datasets available for analysis: the two datasets that we used during model development – the clonal database (training set), and an external set of site-directed mutants that we used for evaluation of mutation pairs for our second order model (validation set) – and two population datasets of clinical isolates: the dataset with samples from which we derived the clones (seen data), and an independent test set (unseen data).

Our results indicated that RAL resistance could be accurately predicted using linear regression modeling.

## Methods

### Clonal INI genotype-phenotype database construction

We derived the Virco clonal INI genotype-phenotype database from 153 clinical isolates, originating from INI naïve and RAL treated patients, including 106 HIV-1 infected patients previously described [13]. Plasma samples were collected before and/or during RAL treatment.

The production of the population recombinant viruses was done as previously described [13]. Briefly, RNA is extracted from plasma and the IN gene is amplified. The replication-competent recombinant virus stocks were produced via homologous recombination in MT4 cells. The purified IN amplicons were recombined within the cells with the pHXB2-ΔIN backbone by Amaxa nucleofection. The cell cultures were microscopically monitored for the appearance of cytopathic effect during the course of infection. When full cytopathic effect was reached, the supernatants containing the recombinant viruses were harvested by centrifugation. For the production of the clonal recombinant viruses, the purified IN amplicons were cloned into the backbone pHXB2-DIN-eGFP using the Clontech In-Fusion technology, following the manufacturer’s protocol. The recombinant plasmids were transformed into Max Efficiency Stbl2 cells (Invitrogen) using the manufacturer’s procedure. Individual clones were randomly picked and cultured to prepare full-length vector HIV-1 genome DNA using the QiaPrep Spin Miniprep system (Qiagen). Replication-competent recombinant virus stocks were generated by nucleofection of full-length HIV-genome plasmids into MT4 cells (Amaxa Biosystems, Cologne, Germany). The cell cultures were microscopically monitored for the appearance of cytopathic effect during the course of infection. When full cytopathic effect was reached, the supernatants containing the recombinant viruses were harvested by centrifugation.

The recombinant viruses were titrated and subjected to an antiviral experiment in MT4-LTR-eGFP cells as previously described [13]. Fold change (FC) values were calculated, using the HIV-1 wild-type strain IIIB as a reference.

Sequence analysis was also done as previously described [13]. Genotypes were defined as a list of IN mutations compared to the HIV-1 wild-type strain HXB2.

In total, our INI genotype-phenotype clonal database consisted for RAL of 991 clonal viruses: 899 clones derived from 153 clinical isolates (93.7% clade B, 6.3% clade non-B), 4 pHXB2D clones and 88 clones derived from 28 site-directed mutants, with a minimum of 2 clones per site-directed mutant. The site-directed mutants incorporated in the clonal database were the ones described in [13]: 66A, 66I, 92Q, 143R, 147G, 148R, 155H, 92Q + 147G, 92Q + 155H, 140S + 148H and 72I + 92Q + 157Q. In addition, site-directed mutants were constructed for IN mutations with score > 0 for RAL/elvitegravir(EVG) in the Stanford algorithm 6.0.11 (http://hivdb.stanford.edu) and either absent in patient derived clones: 66K, 92V, 114Y, 121Y, 125K, 128T, 140C, 143H, 145S, 146P, 151A, 153Y, 155S and 263K or underrepresented: 51Y (1 clone) and 143C (11 clones). Mutation 72A was not found in any of the patient derived clones and it does not appear in the Stanford database of INI resistance mutations (http://hivdb.stanford.edu/DR/INIResiNote.html). Therefore a site-directed mutant, which had been previously created and in vitro had FCs of 1.71 and 4.85 for RAL and EVG, respectively was included in our database. By picking on average 6 clones for each of the 153 clinical isolates and including site-directed mutants, the IN database consisted of 433 unique clonal genotypes.

We calculated a biological cutoff for RAL [14] for the clonal database as the 97.5 percentile of the log FC phenotypes of patient-derived clonal viruses not containing any of the mutations listed in the RAL product label [15]: 74M, 92Q, 97A, 138A/K, 140A/S, 143C/H/R, 148H/K/R, 151I, 155H, 163R, 183P, 226C/D/F/H, 230R and 232N, and/or not containing mutations with score > 0 for RAL/EVG in the Stanford algorithm 6.0.11. Before calculating the biological cutoff, we removed outliers (log FC > mean log FC + 3 standard deviations).

### Consensus linear regression modeling for INI

To perform linear regression on our clonal genotype-phenotype database, we first encoded the clonal genotypes as 0/1 (absence/presence) for all IN mutations present at least once in the database.

We then used a two-stage genetic algorithm (GA) consensus approach to derive a linear regression model for calculating INI resistance (log FC) as the sum of IN mutations or mutation pairs. In stage 1, we ran multiple GA searches to find first order regression models with R^2^ ≥ *goal R*^*2 *^(GA *solutions*). In stage 2, we used a stepwise regression procedure to generate a first order/second order consensus model by considering IN mutations or mutation pairs for entry by descending prevalence in these GA *solutions*.

### Stage 1: Run multiple GAs to select and rank IN mutations

In concept, a GA [16,17] is a computational search procedure where a randomly initialized set (*population*) of encoded genotypes (*chromosomes*) is evolved over several *generations* by optimization of the quality (*fitness*) of the *chromosomes*, and applying *genetic operators* (*mutation* and *crossover*). The GA search is successful once a *chromosome* with *fitness* ≥ *goal fitness* (GA *solution*) is found.

In our application, in search for an INI resistance linear regression model with R^2^ ≥ *goal R*^*2*^, a *chromosome* was a fixed-length subset of IN mutations. The *fitness* of a *chromosome* was evaluated by calculating the R^2^ of the linear model. The implementation of the *genetic operators* was as follows. The *mutation genetic operator* randomly replaced an IN mutation used as linear model parameter by another IN mutation. The *crossover genetic operator* randomly combined two *chromosomes* present within the *population*. In generating a new *population*, the *principle of natural selection* applied: IN mutations present in *chromosomes* that were *more fit* (higher R^2^) had more chance to be selected in a *chromosome* in the next *generation*. To avoid overfitting, we chose the different GA parameter settings such that a *chromosome* reached the *goal fitness* within a limited number of *generations*. As we ran multiple GAs, we could make a ranking of IN mutations based on their prevalence (high to low) in the different GA *solutions*.

For RAL, we performed multiple GA runs until 100 *solutions* were obtained for making a GA ranking. The GAs were run using the R package GALGO [18] with the following settings: *population size* = 20, *chromosome size* = 30, *maximum number of generations* = 500, *goal fitness* = 0.95, *mutation probability* = 0.05 and *crossover probability* = 0.70.

### Stage 2: Run stepwise regression to derive a GA consensus first order/second order model

We derived a consensus first order linear regression model by means of forward stepwise regression, considering IN mutations in order of the GA ranking, and using Schwarz Bayesian Criterion (SBC) for selection. The stepwise procedure ended when SBC reached a minimum [19]. In building the RAL consensus first order linear regression model, we considered mutations that were consistently selected (> 10% prevalence in the GA *solutions*).

To account for synergistic and antagonistic effects between mutations, we allowed mutation pairs (second order interaction terms) of which both mutations in the pair were present in more than T% of the GA models for entry in the model. A threshold of T = 100% corresponded with a first order linear regression model, while lowering T allowed for more interaction terms. For RAL, we chose the threshold T to maximize the R^2 ^performance on a public geno/pheno set of 67 IN site-directed mutants, available from Stanford (http://hivdb.stanford.edu/cgi-bin/IN_Phenotype.cgi), contributed by the following sources: [20] (11 isolates), [21] (14 isolates), [22] (18 isolates), [23] (10 isolates) and [24] (14 isolates). Phenotyping of the isolates in this external geno/pheno set had been done with the PhenoSense assay (Monogram, South San Francisco), providing for validation of the in-house Virco assay. In the stepwise selection procedure, we kept IN mutations as first order terms in the model when also present in a mutation pair.

### Performance evaluation of RAL linear regression model

We analyzed the R^2 ^performance on the clonal database (training set), on the external geno/pheno set (validation set (see previous section)), on the population genotype-phenotype data of the clinical isolates that were used for the clonal database (population seen data), and on population genotype-phenotype data of 171 clinical isolates from RAL treated and INI naïve patients, that were not used for the clonal database (population unseen data). This unseen test set contained clonal genotypes from the three resistance pathways: 143, 148, and 155. We analyzed the performance on population data (seen/unseen) separately for clinical isolates with/without mixtures that contain one or more mutations from the second or first order linear regression model (a mixture is defined as an ambiguous sequencing result at a given amino acid position). To predict the phenotype for isolates containing mixtures, we used equal frequencies for all variants [10]. We also calculated the R^2 ^performance on the clinical isolates with mixtures after removal of outlying samples (having a studentized residual larger than 2 in absolute value). To compare the performance of first and second order models, we used the Hotelling-Williams test [25].

We also used the exact binomial test to calculate the 95% confidence interval for the *true* mixture frequencies from the observed variant frequencies in the clones. We used these mixture frequencies to predict the phenotype for the population seen dataset. In case of more than one mixture in a genotype, we calculated a predicted phenotype for all combinations of lower and upper bounds for the different mixtures. We then plotted the bars of the resulting lowest and highest predicted value.

In the population unseen dataset, we evaluated the linear model biological cutoff call (Susceptible (≤ biological cutoff) or Resistant (> biological cutoff)) versus three public genotypic algorithms: Stanford 6.0.11, Rega v8.0.2 (http://regaweb.med.kuleuven.be/) and ANRS May 2011 (http://www.hivfrenchresistance.org).

## Results

### IN clonal genotype/phenotype database

**Figure 1:**
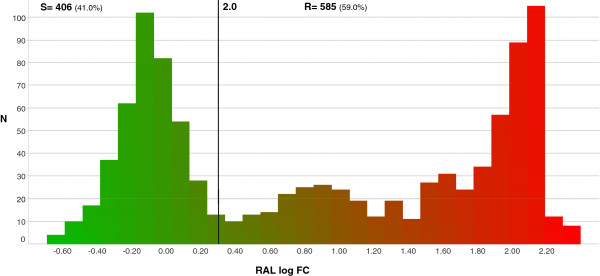
**Phenotype distribution within the INI clonal genotype-phenotype training dataset.** RAL log FC of 991 clones derived from clinical isolates and site-directed mutants. RAL biological cutoff was 0.30 log FC or 2 FC. 41.0% of the clones were found below the biological cutoff and classified as (S)usceptible, whereas 59.0% of the clones were found above the biological cutoff and classified as (R)esistant. Censoring was applied for high FCs.

**Figure 2:**
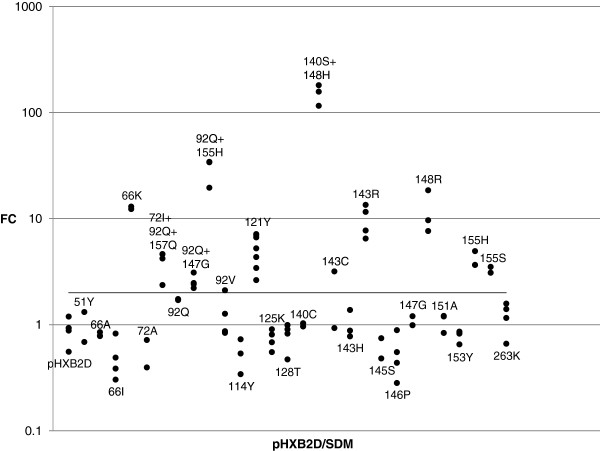
**Phenotypes of wild-type pHXB2D and site-directed mutants.** RAL FC of wild-type pHXB2D (4 clones) and 28 site-directed mutants (88 clones). At least 2 clones were included for each site-directed mutant.

The IN clonal database consisted of 991 clones with genotype and phenotype in log FC for RAL. The distribution of these phenotypes is shown in Figure 1. The biological cutoff for RAL FC was calculated to be 2.0. The calculation was done on 317 clonal viruses with ‘susceptible’ genotypic profile and non-outlying phenotype. This biological cutoff is in agreement with earlier values calculated from INI naïve patient samples [26,27]. The following site-directed mutants that were included in the clonal database had a mean FC above the biological cutoff for RAL: 66K, 72I + 92Q + 157Q, 92Q + 147G, 92Q + 155H, 121Y, 140S + 148H, 143C, 143R, 148R, 155H and 155S (Figure 2).

### RAL linear regression model developed on clonal database

**Figure 3:**
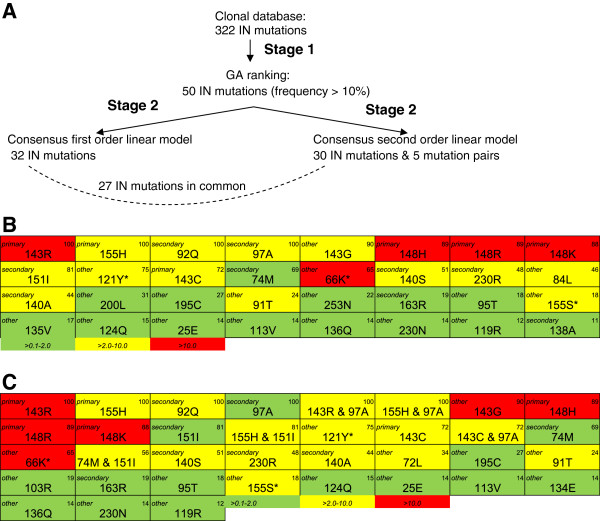
**RAL Linear models. (*A) ***In the clonal genotype-phenotype database, fifty mutations were retained with frequency > 10% in the GA models. Stepwise regression was used for building a first order linear regression model and a second order linear regression model. ***(B) ***First order linear regression model. ***(C) ***Second order linear regression model. In **(*B) ***and ***(C) ***primary and secondary mutations are indicated according to the drug label (upper left corner) and prevalence in GA models is shown (upper right corner). Colouring is based on the FC contribution of the mutations/interaction pairs in the linear regression model: >0.1-2 (green), >2-10 (yellow), >10 (red). *Introduced as site-directed mutant in the model: 66K, 121Y and 155S.

The methodology to develop an INI regression model was tested for RAL. In *generation* 264, the average *fitness* of the 100 GA models reached the *goal fitness*: R^2^ of 0.95. GA runs where the *goal fitness* was not reached with less than 500 *generations* (9.1%) were discarded. As a result of stage 1, fifty mutations out of 322 IN mutations were retained with prevalence above 10% in the GA models (Figure 3). In stage 2, a first order and a second order RAL linear regression model were generated, having 27 IN mutations in common, among which the following primary and secondary RAL product label resistance associated mutations: 143C/R, 148H/K/R and 155H (primary), and 74M, 92Q, 97A, 140A/S, 151I and 230R (secondary). IN mutations present in more than 65 (threshold T) of the 100 GA models were considered for mutation pairs in the second order linear regression model. Five mutation pairs resulted from the stepwise regression procedure: 4 consisting of a primary mutation and a secondary mutation: 143C/R & 97A and 155H & 97A/151I. One mutation pair selected for the model consisted of two secondary mutations: 74M & 151I (Figure 3). We analyzed the frequencies of occurrence of the linear model mutations occurring in first and/or second order linear regression model in the Stanford database for 4240 clinical isolates of INI-naïve (2274 clade B, 1966 clade non-B) and 183 clinical isolates of RAL-treated patients (178 clade B, 5 clade non-B) (http://hivdb.stanford.edu/cgi-bin/II_Form.cgi) (see Additional file 1). R^2 ^performances of the RAL linear model on the training data were 0.96 and 0.97 in first and second order, respectively. On the validation dataset the R^2 ^performance was 0.79 and 0.80 in first and second order, respectively (Table 1). Table 1 also contains the performance on population data, further described in the next sections.

**Table 1:** Performance of RAL first/second order linear model

			**Correlation with measured phenotype (R**^**2**^**)**		**FIRST *vs. *SECOND order (p-value)**
		**N**	**FIRST order**	**SECOND order**	
train	ALL	991	0.96	0.97	0.0019
validation	ALL	67	0.79	0.80	0.3753
population seen	ALL	144^a^	0.90	0.90	0.1530
	NOMIX	105	0.95	0.95	0.3558
	MIX > = 1	39	0.73	0.71	0.0373
	MIX > = 1 (no outliers)	36	0.84	0.81	0.0063
population unseen	ALL	171	0.76	0.78	0.0992
	NOMIX	153	0.79	0.81	0.1358
	MIX > = 1	18	0.59	0.58	0.7482
	MIX > = 1 (no outliers)	16	0.78	0.78	0.8819

^a^For 144 out of 153 samples a matched genotype-phenotype was available.

Performance in R^2 ^of first order and second order linear model on the clonal geno/pheno training database, on the externally PhenoSense measured IN site-directed mutants (validation) and on population data, seen and unseen. After Bonferroni correction for multiple testing no significant differences in R^2 ^performance are seen between the first and second order model on the validation dataset or population seen/unseen datasets (p-values listed are unadjusted).

**Figure 4:**
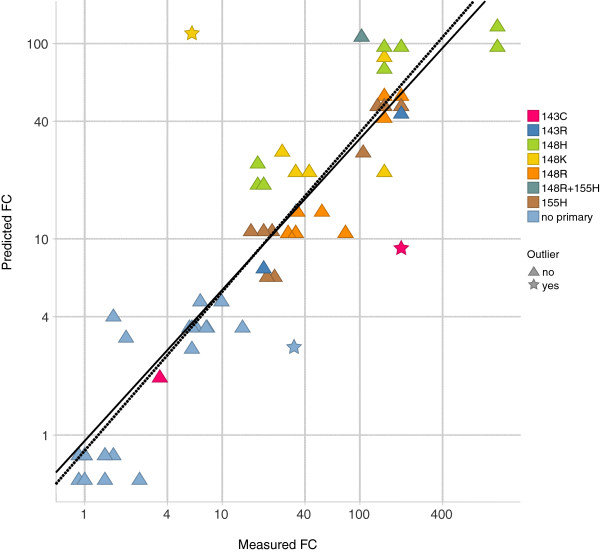
**Performance of RAL second order model on external validation set.** The R^2 ^performance of the RAL second order model on the validation set containing 67 IN site-directed mutants (measured with PhenoSense assay) was 0.80. Three potential outliers were identified: 148K + 140S, 66I + 92Q and 143C + 97A. After outlier removal the R^2 ^performance was 0.91 (dotted regression line). In the graph, site-directed mutants are classified according to the primary mutation harboured.

The R^2^ performance on the validation data improved from 0.80 to 0.91 for the RAL second order linear model after removal of three outliers: 148K + 140S, 66I + 92Q and 143C + 97A (Figure 4). The first and second outlier mutation combination were not present in the clonal database. For the third outlier four clones, derived from one patient, were present.

### Performance of RAL linear regression model on population data (seen)

**Figure 5:**
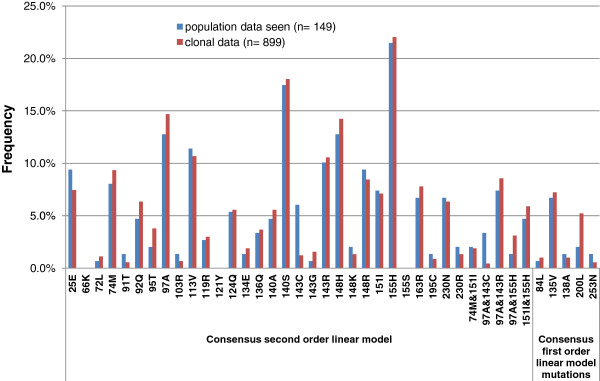
**Prevalence of RAL linear model parameters in clonal *vs. *population genotypes.** Frequency of RAL linear model parameters in clonal and population genotypes retrieved from the same patients. Using Fisher exact test, there are no statistically significant differences between clonal and population percentages, with exception of 143C (P-value < 0.001) and 97A&143C (P-value is 0.004) where clonal frequency is lower.

**Figure 6:**
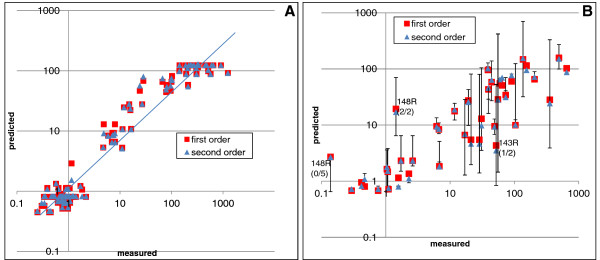
**Performance of RAL linear regression model on population seen data. *(A) ***R^2 ^performance of linear model on patient genotypes without mixtures was 0.95. ***(B) ***Scatterplot of measured FC *vs.* predicted FC for genotypes containing mixtures from 39 clinical isolates. Error bars are drawn to indicate the uncertainty of the second order linear model prediction as the *true* mixture frequencies are unknown. For the three outliers identified, each containing a mixture at a primary mutation in the population genotype, the percentage of clones containing that particular primary mutation was: 0% (0/5, 148R), 50% (1/2, 143R) and 100% (2/2, 148R).

The frequencies of the linear model mutations in the patient-derived clonal genotypes and in the population genotypes for the same patients were largely similar (Figure 5). However, IN mutation 143C was less frequently observed in clones than in the population genotypes, and we made a site-directed mutant for this mutation (Figure 2). The following linear model mutations were not found in any of the patients and appeared in the model as a result of the included site-directed mutants: 66K, 121Y and 155S (Figures 2, 3, 5). The R^2 ^performance of the first order and second order linear model on the population genotypes with measured phenotype was 0.90 (Table 1). The R^2^ performance was analyzed separately for samples with/without mixtures containing linear model mutations. The percentage of samples without mixtures, as detected by population sequencing, was 72.9%. Clonal genotypes were more diverse for the group of clinical isolates with one or more mixtures containing linear model mutations in their population genotype (Table 2). The R^2 ^performance on samples without mixtures was 0.95 in first and second order. The R^2 ^performance on the samples with mixtures was 0.73 and 0.71 in first and second order, respectively and increased to 0.84 and 0.81 after removal of outliers (Table 1 and Figure 6). Although the evaluation with error bars shows that the range of the predicted phenotype due to mixtures containing linear model mutations can be wide, averaging for mixtures resulted overall in a good correlation with the measured phenotype (Figure 6B).

**Table 2:** Diversity of clonal genotypes derived from clinical isolates

**Group (#mixtures in population genotype)**	**#clinical isolates**	**Average #clones/clinical isolate**	**Average #unique clonal genotypes/clinical isolate**	**Percentage of unique clonal genotypes**
0 mixtures	105	5.8	2.4	42.0%
1 mixture	26	5.8	3.8	64.9%
≥ 2 mixtures	13	7.0	4.5	64.8%

Population data seen (144 genotypes, with matched phenotype) divided in groups depending on the number of mixtures containing linear model mutations.

### Performance of RAL linear regression model on population data (unseen)

**Figure 7:**
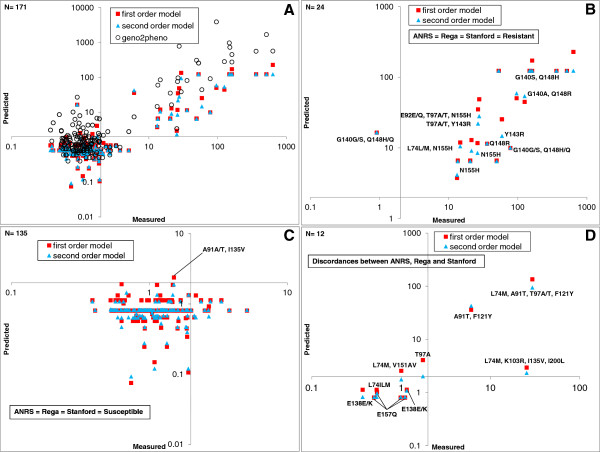
**Performance of RAL linear regression model on population unseen data.** Scatterplot of measured FC *vs.* predicted FC for the first and second order linear model on unseen data. Horizontal and vertical axes are drawn through the biological cutoff value of FC = 2.0. ***(A) ***The R^2 ^performance was 0.76 (first order), 0.78 (second order) and 0.75 (geno2pheno). ***(B) ***Samples called resistant by ANRS, Rega and Stanford are predicted resistant by the linear model. ***(C) ***Samples called susceptible by ANRS, Rega and Stanford are predicted susceptible by the linear model. ***(D) ***Samples with discordant call between the genotypic algorithms.

On the unseen data the R^2 ^performance was 0.76 and 0.78 for the first and second order model, respectively (Table 1, Figure 7A). Eighty-nine percent of the unseen population genotypes had no mixtures containing linear model mutations and had an R^2 ^performance of 0.79 and 0.81 in first and second order, respectively. Using the online prediction tool geno2pheno integrase 2.0 (http://integrase.bioinf.mpi-inf.mpg.de/index.php), the R^2 ^performance was 0.75 and 0.76 on the unseen data and the unseen data without mixtures, respectively. Using the RAL biological cutoff, a resistance call was made for all of the unseen samples. A resistant (R) and susceptible (S) call was given to the samples with linear model prediction above and less or equal than the biological cutoff, respectively. For the samples with a concordant call between ANRS, Rega and Stanford (93% of the samples, Figures 7B and 7C), the first and second order linear model call were in agreement, with exception of one sample (A91A/T, I135V) called resistant by the first order linear model. The remaining 7% of samples with discordance between the genotypic algorithms are given in Figure 7D and Table 3. One third of these discordances contained the IN mutation 157Q, called resistant by ANRS algorithm but susceptible by the first and second order linear model, Stanford and Rega algorithms. Two samples (L74M, V151A/V; T97A) were found to be susceptible by the second order model, but resistant by the first order model. To be precise, the sample T97A had a second order model predicted FC of 2.0, equaling the RAL biological cutoff value. Samples containing the secondary mutations 74M and 97A, were also called intermediate resistant (I) by the Rega algorithm. Other discordances found were related to the IN mutations 121Y (called resistant by the RAL linear model) and 138K (called susceptible by the RAL linear model).

**Table 3:** Samples in the unseen population dataset with discordant call between the genotypic algorithms

	**Genotypic algorithms**						**Linear models**			
	**ANRS (May 2011)**		**Rega v8.0.2**		**Stanford 6.0.11**		**First order**		**Second order**	
Sample^a^	mutations	call	mutations	call	mutations	call	mutations	BCO call	mutations	BCO call
L74ILM	72I, 74M	S	74M, 156N	I	74M	S	74M	S	74M	S
L74M, A91 T, T97A/T, F121Y	72I, 74M, 97A, 121Y	R	74M, 97A, 206S	I	74M, 97A, 121Y	I	74M, 91T, 97A, 119R, 121Y	R	74M, 91 T, 97A, 119R, 121Y	R
L74M, K103R, I135V, I200L	72I, 74M	S	74M	I	74M	S	74M, 135V, 200L	R	74M, 103R	R
L74M, V151A/V	72I, 74M	S	74M	I	74M, 151A	S	74M, 135V, 200L	R	74M, 103R	S
A91 T, F121Y	72I, 121Y	R	206S	S	121Y	I	91 T, 119R, 121Y	R	91T, 119R, 121Y	R
T97A	72I, 97A	S	97A, 206S	I	97A	S	25E, 97A	R	25E, 97A	S
E138E/K	72I	S	138K, 206S	S	138K	I	135V	S	-	S
E138E/K	-	S	138K	S	138K	I	25E	S	25E	S
E157Q	72I, 157Q	R	156N	S	157Q	S	-	S	-	S
E157Q	72I, 157Q	R	206S	S	157Q	S	135V	S	-	S
E157Q	157Q	R	-	S	157Q	S	-	S	-	S
E157Q	157Q	R	156 N, 206S	S	157Q	S	-	S	-	S

^a^HXB2 reference amino acid is shown.

The mutations on which the RAL S(usceptible)/I(ntermediate)/R(esistant) call is based, are listed. For the linear models, the call is R(esistant) if FC prediction is above the RAL biological cutoff (BCO = 2.0).

## Discussion

We developed a methodology for predicting INI susceptibility, applying linear regression on a clonal genotype-phenotype database. Our modeling approach differs from most of the other genotypic INI resistance interpretation systems by providing a quantitative FC prediction. A particular advantage of our model is that predictions can be directly interpreted as a weighted sum of mutations and interaction pairs. We have made our RAL second order linear regression model available as PDF fillable form in Additional file 2 such that it can be used for rapid prediction of RAL susceptibility.

Previously, we described a computationally feasible technique for developing parsimonious linear regression models on large genotype-phenotype datasets for the identification of novel HIV-1 drug resistance associated mutations [28]. In this article, as the number of patients failing INI treatment was limited, our primary objective was to develop a methodology for training a linear regression model on a relatively small dataset. We increased the quality of the correlative genotype-phenotype data by taking multiple clones for each of the clinical isolates [26], allowing to more accurately model the resistance contribution of IN mutations or mutation pairs. Moreover, to avoid overfitting, we generated an INI model by consensus linear regression modeling, using a GA for selection of IN mutations [29,30]. Multiple clones taken from the same patient largely confirmed the independence of the RAL resistance pathways 143, 148 and 155 [24,31,32]. For one patient, previously described in [33], four clones were picked containing both 143C and 155H. Mutation 143C was found to have a low prevalence in the clonal database. In [34] a transition from 143C to 143R was suggested, and in our RAL linear model 143R had a larger contribution towards resistance than 143C. 143G was another resistance associated variant at position 143 selected for our linear model, and has been described in [35,36]. Obviously, our approach is still limited to detecting resistance associated mutations or combinations of mutations with presence in the training dataset. This was in part overcome by inclusion of site-directed mutants in the analysis, which we consider valuable in improving the generalizability of the model.

We evaluated the performance of the RAL linear model on an unseen population dataset. For RAL, the additive first order model had an overall equal performance to the second order model, which accounted for synergism or antagonism. However, for an individual sample (T97A) with secondary mutation 97A, found in absence of a primary mutation, a discordance was seen between the first and second order linear models. It was scored resistant by the first order model and susceptible by the second order model when using a biological cutoff of 2. In two other samples (T97A/T, Y143R; E92E/Q, T97A/T, N155H) where primary mutations 143R or 155H occurred together with 97A (in mixture with wild type), the increased resistance conferred by the combinations 143C/R & 97A [37] or 155H & 97A, was in the second order model accounted for by interaction terms. Because the second order model explicitly includes combination effects, we consider it more useful than the first order model. All interaction terms in the second order model were found to be synergistic. A high concordance in RAL resistance call was seen between the linear model and the publically available genotypic algorithms: Stanford, Rega and ANRS. However, major discordances were observed for samples without a primary mutation and containing mutation 157Q or 121Y. For the discordance involving 157Q, already discussed in [38], four clinical isolates (E157Q) from different patients were called Susceptible by the linear model, Stanford and Rega, but Resistant by ANRS. For the discordance involving 121Y, one clinical isolate (A91T, F121Y) was called Resistant by the linear model and ANRS, Intermediate resistant by Stanford, but Susceptible by Rega. According to [11], the in vivo selection of 121Y has not yet been reported. In the current study, one patient was found in the unseen dataset, who had indeed developed the 121Y mutation. However, as 121Y was not observed in any of the patient derived clones for training of the linear model, we had made seven site-directed mutant clones for the clonal genotype-phenotype database, confirming the in vitro effect of 121Y [7] on RAL resistance. As a result, 121Y could be and was selected for the linear model, and contributed to the FC prediction of the two clinical isolates from the aforementioned patient. Note that in the genotype of these isolates also the rare mutation 91T was found, a mutation that has not been associated with RAL resistance, but contributed to resistance in the RAL linear model. From the unseen data, it seems as if 91T may be a background mutation that is currently overweighted in the linear model. However, more samples are needed to be conclusive about 91T.

Other rare mutations in the RAL linear model that needed to be inspected more carefully were 72L and 84L, as they are currently undescribed and contributed to resistance in the second and first order model, respectively. Remarkably, 72L and 84L co-occurred in the clonal genotypes of nine clinical isolates derived from a single patient (only 72L appeared in another clinical isolate, by itself). In the clones of this patient the secondary mutations 74M, 92Q and 151I were also found, in absence of any primary mutations, and the measured RAL FCs were above the biological cutoff (42.9–77.4). Thus, although 72L and/or 84L are potential RAL resistance associated mutations, it may be possible that resistance for this patient is explained by a more complex synergistic interaction between 74M, 92Q and 151I. Note that mutation pair 74M & 151I had been selected for the RAL second order linear model, which already indicates that INI resistance can be developed between interacting secondary mutations, in absence of a primary mutation. Moreover, interactions between mutations are expected to become more important in elucidating genotype-INI susceptibility phenotype relationships once several INIs will be co-administered.

When comparing the R^2 ^performance of the RAL linear model on population data, unseen *vs.* seen, a lower R^2 ^performance on unseen data was observed. This difference in performance was acceptable as in the unseen dataset there were more clinical isolates that did not contain any of the primary RAL resistance mutations in their genotype (82.5% *vs.* 45.0%), and the measurement error of the phenotypic assay was relatively larger for low FC values.

In the described approach, ordinary least squares regression (OLS) was used without taking into account the correlation between genotypes-phenotypes of clones from the same clinical isolate or site-directed mutant. One way to account for such correlation would be to replace OLS by a linear mixed model with as *fixed effects* the linear model mutations and mutation pairs as in the RAL second order linear model (Figure 3), and with the clinical isolate/site-directed mutant as random factor. The predictive performance of the resulting model in terms of R^2 ^changed from 0.80 to 0.82 and from 0.78 to 0.79, on the external validation set, and population unseen dataset, respectively. Such a minor change was not unexpected since OLS parameter estimates are known to be unbiased, even when the correlation structure is neglected [39]. Nevertheless, for future work it could be beneficial in using a mixed model instead of OLS for the GA models to improve the selection of the mutations and mutation pairs.

In conclusion, RAL resistance could be estimated using linear regression modeling and produced results that were generally consistent with those observed for samples analyzed by Stanford, Rega and ANRS algorithms or the online prediction tool geno2pheno. The quality of the INI susceptibility models is improved by developing the models on a clonal genotype-phenotype database and using a GA consensus approach. A quantitative linear model predicted phenotype is interpretable and informative about the effect of combinations of mutations on INI resistance. The linear regression modeling approach allows generating reliable models for INIs once viral isolates have been obtained during or after selective pressure of these INIs, even for relatively small numbers of patients.

## Consent

All patient data used in our manuscript were obtained from different collaborators. With each of these collaborators a contract was signed stipulating that patient consent was available from local IRB and/or the competent IRB/EC authorizations were obtained to provide us with the patient samples for research purposes.

## Competing interests

The authors declare that they have no competing interests.

## Authors’ contributions

KVdB designed the study, performed the analysis and drafted the manuscript. AV, MF, LVW and YV were involved in acquisition of samples, lab experiments and preparation of the genotype-phenotype database. EVC and HvV conceived of the study and participated in its design. All authors read and approved the final manuscript.

## Supplementary Material

Additional file 1 **Prevalence of RAL first order/second order linear model mutations in Stanford database.** Frequency of linear model mutations in INI naïve *vs.* RAL treated patients and clade B *vs.* non-B. Click here for file

Additional file 2 **RAL second order linear model.** The TGZ archive file contains the following two files. In PDF file is the Raltegravir Resistance Phenotype Linear Model Prediction Tool. Users can indicate linear model mutations present in the IN sequence of a clinical isolate as well as the presence of more than one variant (mixture) at a resistance position. In calculating RAL resistance, a weighted sum (in log FC) is made of the linear model IN mutations and mutation pairs present in the isolate, applying averaging for mixtures. In TXT file, the linear model coefficients are given together with their P-values and 95% Confidence Intervals. Click here for file

## References

[B1] Adamson CS Freed EO Recent progress in antiretrovirals – lessons from resistance Drug Discov Today 2008 13 424 432 10.1016/j.drudis.2008.02.003 18468560 PMC2581412

[B2] Flexner C HIV drug development: the next 25 years Nat Rev Drug Discov 2007 6 959 966 10.1038/nrd2336 17932493

[B3] Marchand C Maddali K Métifiot M Pommier Y HIV-1 IN inhibitors: 2010 update and perspectives Curr Top Med Chem 2009 9 1016 1037 10.2174/156802609789630910 19747122 PMC2860603

[B4] McColl DJ Chen X Strand transfer inhibitors of HIV-1 integrase: bringing IN a new era of antiretroviral therapy Antiviral Res 2010 85 101 118 10.1016/j.antiviral.2009.11.004 19925830

[B5] Hicks C Gulick RM Raltegravir: the first HIV type 1 integrase inhibitor Clin Infect Dis 2009 48 931 939 10.1086/597290 19231980

[B6] Nguyen BY Isaacs RD Teppler H Leavitt RY Sklar P Iwamoto M Wenning LA Miller MD Chen J Kemp R Xu W Fromtling RA Vacca JP Young SD Rowley M Lower MW Gottesdiener KM Hazuda DJ Raltegravir: the first HIV-1 integrase strand transfer inhibitor in the HIV armamentarium Ann N Y Acad Sci 2011 1222 83 89 10.1111/j.1749-6632.2011.05972.x 21434946

[B7] Kobayashi M Yoshinaga T Seki T Wakasa-Morimoto C Brown KW Ferris R Foster SA Hazen RJ Miki S Suyama-Kagitani A Kawauchi-Miki S Taishi T Kawasuji T Johns BA Underwood MR Garvey EP Sato A Fujiwara T In Vitro antiretroviral properties of S/GSK1349572, a next-generation HIV integrase inhibitor Antimicrob Agents Chemother 2011 55 813 821 10.1128/AAC.01209-10 21115794 PMC3028777

[B8] Panel on Antiretroviral Guidelines for Adults and Adolescents, Department of Health and Human Services Guidelines for the use of antiretroviral agents in HIV-1-infected adults and adolescents 2011 [http://www.aidsinfo.nih.gov/ContentFiles/AdultandAdolescentGL.pdf]

[B9] Cortez KJ Maldarelli F Clinical management of HIV drug resistance Viruses 2011 3 347 378 10.3390/v3040347 21994737 PMC3185705

[B10] Vermeiren H Van Craenenbroeck E Alen P Bacheler L Picchio G Lecocq P Prediction of HIV-1 drug susceptibility phenotype from the viral genotype using linear regression modeling J Virol Methods 2007 145 47 55 10.1016/j.jviromet.2007.05.009 17574687

[B11] Blanco JL Varghese V Rhee SY Gatell JM Shafer RW HIV-1 integrase inhibitor resistance and its clinical implications J Infect Dis 2011 203 1204 1214 10.1093/infdis/jir025 21459813 PMC3069732

[B12] Métifiot M Marchand C Maddali K Pommier Y Resistance to integrase inhibitors Viruses 2010 2 1347 1366 10.3390/v2071347 20706558 PMC2920056

[B13] Van Wesenbeeck L Rondelez E Feyaerts M Verheyen A Van der Borght K Smits V Cleybergh C De Wolf H Van Baelen K Stuyver LJ Cross-resistance profile determination of two second-generation HIV-1 integrase inhibitors using a panel of recombinant viruses derived from raltegravir-treated clinical isolates Antimicrob Agents Chemother 2011 55 321 325 10.1128/AAC.01733-09 20956600 PMC3019647

[B14] Verlinden Y Vermeiren H Lecocq P Bacheler L McKenna P Vanpachtenbeke M Horvat LI Van Houtte M Stuyver LJ Assessment of the antivirogram® performance over time including a revised definition of biological test cut-off values Antivir Ther 2005 10 S51

[B15] FDA Isentress (raltegravir) drug label 2009 [http://www.accessdata.fda.gov/drugsatfda_docs/label/2009/022145s004lbl.pdf]

[B16] Holland JH Adaptation in natural and artificial systems 1975 Ann Arbor: University of Michigan Press

[B17] Goldberg DE Genetic algorithms in search, optimization and machine learning 1989 Reading, MA: Addison-Wesley

[B18] Trevino V Falciani F GALGO: an R package for multivariate variable selection using genetic algorithms Bioinformatics 2006 22 1154 1156 10.1093/bioinformatics/btl074 16510496

[B19] Kutner MH Nachtsheim CJ Neter J Li W Applied Linear Statistical Models 2004 New York: McGraw-Hill

[B20] Jones G Ledford R Yu F Miller M Tsiang M McColl DJ Resistance profile of HIV-1 mutants in vitro selected by the HIV-1 integrase inhibitor, GS-9137 (JTK-303) 2007 Los Angeles, CA: 14th Conference on Retroviruses and Opportunistic Infections

[B21] McColl DJ Fransen S Gupta S Parkin N Margot N Ledford R Chen J Chuck S Cheng AK Miller D Resistance and cross-resistance to first generation integrase inhibitors: insights from a Phase II study of elvitegravir (GS-9137) Antivir Ther 2007 12 S11

[B22] Goodman D Hluhanich R Waters J Margot NA Fransen S Gupta S Huang W Parkin N Borroto-Esoda K Svarovskaia ES Miller MD McColl DJ Integrase inhibitor resistance involves complex interactions among primary and secondary resistance mutations: a novel mutation L68V/I associates with E92Q and increases resistance Antivir Ther 2008 13 A15

[B23] Fransen S Gupta S Frantzell A Petropoulos C Huang W HIV-1 mutations at positions 143, 148, and 155 of integrase define different genetic barriers to raltegravir resistance in vitro 2009 Montreal, Canada: 16th Conference on Retroviruses and Opportunistic Infections

[B24] Fransen S Gupta S Danovich R Hazuda D Miller M Witmer M Petropoulos CJ Huang W Loss of raltegravir susceptibility by human immunodeficiency virus type 1 is conferred via multiple nonoverlapping genetic pathways J Virol 2009 83 11440 11446 10.1128/JVI.01168-09 19759152 PMC2772690

[B25] Steiger JH Tests for comparing elements of a correlation matrix Psychol Bull 1980 87 245 251

[B26] Van Baelen K Rondelez E Van Eygen V Ariën K Clynhens M Van den Zegel P Winters B Stuyver LJ A combined genotypic and phenotypic human immunodeficiency virus type 1 recombinant virus assay for the reverse transcriptase and integrase genes J Virol Methods 2009 161 231 239 10.1016/j.jviromet.2009.06.015 19559730

[B27] Rondelez E Van Baelen K Ceccherini-Silberstein F Van Eygen V Van den Zegel P Winters B Armenia D Trignetti M Perno CF Stuyver LJ Preliminary biological cutoffs for GS-9137 and MK-0518 integrase inhibitors derived from clonal phenotypic analysis 2008 Budapest, Hungary: 6th European HIV Resistance Workshop

[B28] Van der Borght K Van Craenenbroeck E Lecocq P Van Houtte M Van Kerckhove B Bacheler L Verbeke G van Vlijmen H Cross-validated stepwise regression for identification of novel non-nucleoside reverse transcriptase inhibitor resistance associated mutations BMC Bioinformatics 2011 12 386 10.1186/1471-2105-12-386 21966893 PMC3223907

[B29] Leardi R Boggia R Terrile M Genetic algorithms as a strategy for feature selection J Chemom 1992 6 267 281 10.1002/cem.1180060506

[B30] Sudjianto A Wasserman GS Sudarbo H Genetic subsets regression Computers Ind Engng 1996 30 839 849 10.1016/0360-8352(95)00182-4

[B31] Reigadas S Anies G Masquelier B Calmels C Stuyver LJ Parissi V Fleury H Andreola ML The HIV-1 integrase mutations Y143C/R are an alternative pathway for resistance to Raltegravir and impact the enzyme functions PLoS One 2010 5 e10311 10.1371/journal.pone.0010311 20436677 PMC2859942

[B32] Quercia R Dam E Perez-Bercoff D Clavel F Selective-advantage profile of human immunodeficiency virus type 1 integrase mutants explains in vivo evolution of raltegravir resistance genotypes J Virol 2009 83 10245 10249 10.1128/JVI.00894-09 19605484 PMC2747997

[B33] Ceccherini-Silberstein F Armenia D D’Arrigo R Vandenbroucke I Van Marck H Van Baelen K Van Wesenbeeck L Rizzardini G Lo Caputo S Narciso A Stuyver L Perno CF Primary mutations associated with resistance to raltegravir are not detectable by pyrosequencing in integrase-inhibitors naïve patients 2010 San Francisco, CA: 17th Conference on Retroviruses and Opportunistic Infections

[B34] Delelis O Thierry S Subra F Simon F Malet I Alloui C Sayon S Calvez V Deprez E Marcelin AG Tchertanov L Mouscadet JF Impact of Y143 HIV-1 integrase mutations on resistance to raltegravir in vitro and in vivo Antimicrob Agents Chemother 2010 54 491 501 10.1128/AAC.01075-09 19901095 PMC2798554

[B35] Huang W Fransen S Frantzell A Petropoulos C Identification of alternative amino acid substitutions at HIV-1 integrase codon 143 that confer reduced susceptibility to RAL 2011 Boston, MA: 18th Conference on Retroviruses and Opportunistic Infections

[B36] Tsurutani N Kubo M Maeda Y Ohashi T Yamamoto N Kannagi M Masuda T Identification of critical amino acid residues in human immunodeficiency virus type 1 IN required for efficient proviral DNA formation at steps prior to integration in dividing and nondividing cells J Virol 2000 74 4795 4806 10.1128/JVI.74.10.4795-4806.2000 10775618 PMC112002

[B37] Reigadas S Masquelier B Calmels C Laguerre M Lazaro E Vandenhende M Neau D Fleury H Andréola ML Structure-analysis of the HIV-1 integrase Y143C/R raltegravir resistance mutation in association with the secondary mutation T97A Antimicrob Agents Chemother 2011 55 3187 3194 10.1128/AAC.00071-11 21576445 PMC3122421

[B38] Wiesmann F Braun P Van Houtte M Voigt E Ehret R Van Wesenbeeck L Knechten H HIV-1 integrase mutation E157Q has low impact on integrase inhibitor resistance: a case report Rev Antivir Ther Infect Dis 2010 1 33

[B39] Verbeke G Molenberghs G Linear mixed models for longitudinal data 2000 New York: Springer

