# Supplementary material for: Quantitative prediction of integrase inhibitor resistance from genotype through consensus linear regression modeling
Source: Virol J. 2013 Jan 3;10:8. doi: 10.1186/1743-422X-10-8 (PMC3551713; doi:10.1186/1743-422X-10-8)
Supplement: Additional file 1 — Prevalence of RAL first order/second order linear model mutations in Stanford database. Frequency of linear model mutations in INI naïve vs. RAL treated patients and clade B vs. non-B. [file 1743-422X-10-8-S1.pdf]

Prevalence of RAL linear model mutations in Stanford database

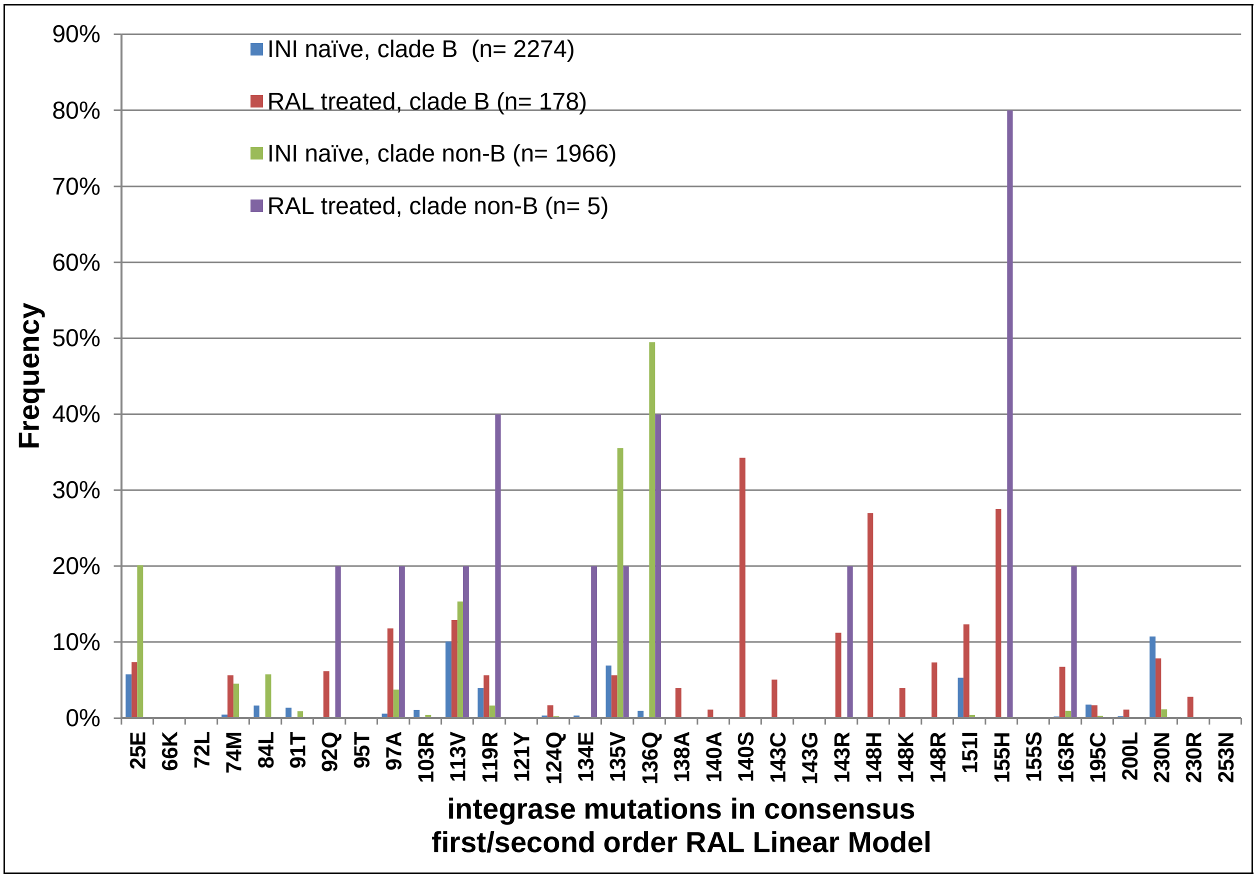

Frequency of linear model mutations in INI naïve *vs.* RAL treated patients and clade B *vs.* non-B.

Secondary resistance associated mutations that were not found in INI naïve patients were 92Q and 140A. Secondary resistance associated mutations with prevalence >3-5% in INI naïve patients were 151I in clade B patients and 74M and 97A in clade non-B patients.
